# Draft genome sequence of *Pseudomonas aeruginosa* strain NGMBE-25A isolated from fecal samples of migratory birds at Jahangirnagar University, Bangladesh

**DOI:** 10.1128/mra.01160-24

**Published:** 2025-06-09

**Authors:** Md. Mashiur Rahaman, Sumaiya Akter, Shoheli Asrafie, Zan Zan U, Raiyan Rafsan, Abdus Sadique, Jahidul Alam, Fahad Khan, Nayma Haque Tonny, Rozely Hossain, Mohammad Moshiur Rahman, Abu Noman Zisan, Md. Baki Billah, Kabir M. Uddin, Md. Mahbubur Rahman, Oly Ahmed, Fariza Shams, Ayesha Sharmin, Md. Mainul Hossain, Maqsud Hossain

**Affiliations:** 1 NSU Genome Research Institute (NGRI), North South University 54495, Dhaka, Bangladesh; 2 Department of Biochemistry & Microbiology, School of Health and Life Sciences, North South University 54495, Dhaka, Bangladesh; 3 Department of Electrical and Computer Engineering, School of Health and Life Sciences, North South University 54495, Dhaka, Bangladesh; 4 Department of Environmental Science and Management, School of Health and Life Sciences, North South University 54495, Dhaka, Bangladesh; 5 Department of Zoology, Jahangirnagar University 115523, Savar, Dhaka, Bangladesh; 6 Department of Pharmaceutical Science, School of Health and Life Sciences, North South University 54495, Dhaka, Bangladesh; 7 Department of Chemistry, Bangladesh University of Engineering and Technology 61750, Dhaka, Bangladesh; California State University San Marcos, San Marcos, California, USA

**Keywords:** *Pseudomonas aeruginosa*, migratory birds, antibiotic resistance genes, pathogen dissemination, environmental microbiology

## Abstract

We report the draft genome sequence of *Pseudomonas aeruginosa* from migratory birds at Jahangirnagar University, Bangladesh. The 6.27 Mb genome, sequenced using Illumina MiSeq, contains 5,719 coding sequences, including antibiotic resistance genes. This report highlights the role of wildlife in the dissemination of bacterial pathogens.

## ANNOUNCEMENT

*P. aeruginosa* is an opportunistic pathogen known for its genetic diversity and adaptability in various environments that include hospital settings and natural reservoirs such as soil, water, and animals (1). It is a major cause of infections in immunocompromised individuals and is commonly associated with antibiotic resistance (2).

We are announcing here the draft genome sequence of *P. aeruginosa* isolated from the migratory bird, Mallard duck, locally known as “Nilshir” (*Anas platyrhynchos*) in Jahangirnagar University (23.8825°N, 90.2482°E) (3), Bangladesh. A fresh drop from the bird was aseptically collected using a sterile cotton swab, placed into 9 mL of LB broth as enrichment medium, and incubated at 37°C for 6 h under aerobic conditions. The culture was then streaked onto MacConkey agar plates and incubated at 37°C for 20 h. A single, well-isolated colony with no visible contamination was selected for further analysis. All animal handling and sampling procedures were conducted in accordance with IACUC approval no. 2025/OR-NSU/IACUC/0210. DNA was extracted from a single colony grown overnight in LB broth, incubated at 37°C using the QIAGEN Mini Kit (QIAGEN, Germany), and the species was confirmed using PCR with *P. aeruginosa*-specific primers (4).

The genome was sequenced on an Illumina MiSeq platform using MiSeq Reagent Kit V3 (Illumina Inc. USA) with a paired-end sequencing (2 × 150 bp) approach that generated 817,109 paired-end reads at NSU Genome Research Institute (NGRI). The library preparation was performed using the Nextera XT Library Preparation Kit (Illumina, USA). Raw reads were processed with FastQC v0.74 (5) for quality assessment, and low-quality reads and adapters were removed using Trimmomatic v0.39 (6) (AVGQUAL 30). After trimming, 789,387 high-quality reads were obtained for genome assembly. *De novo* assembly was performed using Unicycler v0.5.1 (7), resulting in a draft genome with 95 contigs. The total genome length is 6,273,665 bp with a GC content of 66.4%. The N50 value is 141,704 bp with contig lengths ranging from 392,176 bp to 224 bp. The genome was annotated using the Prokaryotic Genome Annotation Pipeline (PGAP) v6.8 by NCBI (8), which showed the genome to contain 5,809 protein-coding genes, 3 rRNAs, and 58 tRNAs. Further downstream processing of the assembled genome in ResFinder v4.7.2 (9, 10), PathogenFinder2 (11), and MLST v2.0 (12) revealed the bacteria to harbor six AMR genes (aph(3')-Iib, blaOXA-50, blaPAO, blaPAO, fosA, and catB7), a potential pathogen to humans with a score of 0.9863 and belonging to the sequence type 782, respectively. Default parameters were used for all tools except where otherwise noted.

## Data Availability

The sequence reads are deposited in the Sequence Read Archive under the accession number SRR31009932 and the assembled genome under the accession JBIOAX000000000. The associated Bioproject and BioSample accession numbers are PRJNA1173360 and SAMN44302774 respectively. The output files from ResFinder, PathogenFinder, and MLST analyses have been made publicly accessible via Figshare at the following DOI: https://doi.org/10.6084/m9.figshare.28844606.v3.
